# Salivary biomarkers for the diagnosis of gastric cancer: a systematic review

**DOI:** 10.21142/2523-2754-1202-2024-199

**Published:** 2024-06-27

**Authors:** Natalia Zúñiga-Pérez, Humberto Muñoz-Navarro, César Rivera

**Affiliations:** 1 Oral and Maxillofacial Histopathology Laboratory, Department of Stomatology, Faculty of Dentistry, Universidad de Talca. Talca, Chile. zuniga.natalia.1997@gmail.com , munozhumberto300@gmail.com , cerivera@utalca.cl Universidad de Talca Oral and Maxillofacial Histopathology Laboratory, Department of Stomatology Faculty of Dentistry Universidad de Talca Talca Chile zuniga.natalia.1997@gmail.com munozhumberto300@gmail.com cerivera@utalca.cl

**Keywords:** saliva, salivary proteins and peptides, biomarkers, tumor biomarkers, stomach neoplasms, saliva, proteínas y péptidos salivales, biomarcadores, biomarcadores tumorales, neoplasias gástricas

## Abstract

**Background:**

Recent advancements reveal saliva as a crucial source of diagnostic biomarkers for various diseases, notably gastric cancer. This systematic review evaluates these biomarkers, emphasizing their clinical applicability and potential in early detection.

**Methods:**

An extensive electronic search was conducted across PubMed/MEDLINE, Scopus, and Web of Science to identify relevant studies. Salivary biomarkers were analyzed as independent variables, with gastric cancer as the dependent variable. The study adhered to a protocol registered with PROSPERO (CRD42021259519).

**Results:**

Our analysis identified a range of biomarkers, highlighting three proteins - cystatin-B (CSTB), triosephosphate isomerase (TPI1), and deleted in malignant brain tumors 1 protein (DMBT1) - as particularly accurate for gastric cancer diagnosis.

**Conclusions:**

Salivary biomarkers hold substantial promise for the early detection of gastric cancer. Future research should aim to refine study design and validation for enhancing the quality and applicability of these biomarkers.

## RESUMEN

**Introducción:**

Avances recientes revelan la saliva como una fuente crucial de biomarcadores diagnósticos para diversas enfermedades, especialmente el cáncer gástrico. Esta revisión sistemática evalúa estos biomarcadores, con énfasis en su aplicabilidad clínica y potencial para la detección temprana.

**Métodos:**

Se realizó una extensa búsqueda electrónica en PubMed/MEDLINE, Scopus y Web of Science para identificar estudios relevantes. Los biomarcadores salivales fueron analizados como variables independientes, con el cáncer gástrico como variable dependiente. El estudio siguió un protocolo registrado en PROSPERO (CRD42021259519).

**Resultados:**

Nuestro análisis identificó una gama de biomarcadores entre los que destacan tres proteínas: cistatina-B (CSTB), triosa fosfato isomerasa (TPI1) y proteína 1 eliminada en tumores cerebrales malignos (DMBT1), como particularmente precisas para el diagnóstico del cáncer gástrico.

**Conclusiones:**

: Los biomarcadores salivales tienen un gran potencial para la detección temprana del cáncer gástrico. La investigación futura debería apuntar a refinar el diseño del estudio y la validación para mejorar la calidad y aplicabilidad de estos biomarcadores.

## INTRODUCTION

Gastric cancer, predominantly adenocarcinoma, represents a significant health challenge. This malignant neoplasm arises from the stomach's lining ^1^ and is known for its propensity to metastasize rapidly to nearby organs ^2^. Accounting for 95% of gastric cancers, adenocarcinoma is classified based on its invasion depth: early stage when confined to the submucosa and advanced stage upon deeper penetration ^2^^,^^3^. The development of this disease is influenced by a variety of factors including genetic predisposition, aging, lifestyle habits such as excessive alcohol intake and smoking, and diets rich in sodium, red meats, and salt-preserved or smoked foods ^4^^,^^5^. A notable contributor, *Helicobacter pylori* infection, is implicated in approximately 75% of gastric cancer cases ^6^. Globally, gastric cancer ranks as the fifth most prevalent malignancy and the fourth leading cause of cancer mortality ^4^, according to GLOBOCAN 2020.

The high mortality rate associated with gastric cancer is largely attributed to delayed diagnosis. Often, the disease remains asymptomatic during initial stages, with symptoms like weight loss, dysphagia, dyspepsia, nausea, vomiting, and early satiety emerging only in advanced stages ^7^. Early detection significantly improves prognosis, with favorable outcomes observed in cases where surgical intervention is possible. In contrast, late-stage diagnosis often results in a survival period as short as six months ^8^. Currently, the definitive diagnosis of gastric cancer involves upper digestive endoscopy followed by a biopsy ^2^, a procedure that is invasive, uncomfortable, and necessitates specialized expertise ^9^.

The pursuit of early gastric cancer detection presents a formidable clinical challenge. Success in this area could dramatically enhance treatment options and patient prognosis. To this end, the investigation of various biomarkers in bodily fluids, including saliva, has gained momentum. Saliva, a complex fluid harboring components like mRNA, microRNAs, and enzymes, reflects both local and systemic health conditions ^10^^,^^11^ and offers a non-invasive, safe, and cost-effective alternative for diagnostic sampling ^12^.

However, despite extensive research, clinically reliable salivary biomarkers for early gastric cancer detection remain elusive. Addressing this gap, our systematic literature review aimed to identify, evaluate, and compare salivary biomarkers nearing clinical application. We focused on studies in their validation phase reporting diagnostic accuracy. This approach led us to identify biomarkers across a spectrum of molecular types, with CSTB, TPI1, and DMBT1 proteins demonstrating remarkable diagnostic precision in independent validation settings.

## METHODS

In this study, we conducted a systematic review. Salivary biomarkers, serving as the independent variables, while the presence of gastric cancer was established as the dependent variable. A protocol was registered with the International Prospective Register of Systematic Reviews (PROSPERO) and assigned the registration code CRD42021259519. 

Diagnostic biomarker: We defined a diagnostic biomarker as a molecule obtained from studies involving binary logistic regression analysis or Cox proportional hazards modeling, showing a statistically significant association between the biomarker and the outcome (gastric cancer). The calculated risk (odds ratio, OR, or hazard ratio, HR) should have been reported as the risk of a specific outcome in the biomarker group compared to the reference group, with OR/HR > 1 indicating higher risk and OR/HR <1 indicating reduced risk ^13^.

Search strategy: We conducted a systematic search of all English and Spanish literature in MEDLINE, Scopus, and Web of Science, up to September 20, 2023. Our search strategy resulted from the combination of the following keywords (Table 1).

**Table 1:** Databases and searches used in this research.

Database	Query
Pubmed/MEDLINE	(("Biomarkers"(Mesh) OR "Biomarkers, Tumor/metabolism"(Mesh) OR "Biomarkers, Tumor"(Mesh)) AND ("Saliva"(Mesh)) AND ("Stomach Neoplasms"(Mesh) OR Gastric cancer (all fields)))
Scopus	(((("Biomarkers" OR "Biomarkers, Tumor/metabolism" OR "Biomarkers, Tumor") AND ("Saliva")) AND ("Stomach Neoplasms" OR “Gastric cancer”)))
Web of Science	TS=”Biomarker*”AND TS=”Saliva“ AND TS=(”Stomach Neoplasms” OR ”Gastric cancer“) AND TS=”Diagnosis”

Data extraction: Two review authors independently extracted the data in duplicate. Disagreements were resolved with the intervention of a third investigator, who made the final decision. The following data were extracted from each study: complete reference, study characteristics, country, ethnic origin, sample size (cancer vs. non-cancer), cancer type, type of saliva (stimulated or unstimulated), saliva sample (whole saliva, supernatant, or sediment), and diagnostic capacity values.

Inclusion and exclusion criteria: We included observational studies (prospective and retrospective cohort studies, case-control studies, cross-sectional studies) that reported diagnostic accuracy values of salivary biomarkers in gastric cancer. We excluded preliminary studies, conference abstracts, and literature reviews presented in languages other than English or Spanish. After full-text analysis, we excluded studies that did not present an independent validation phase.

Quality assessment: The Quality Assessment of Diagnostic Accuracy Studies 2 (QUADAS-2) checklist was used ^14^. Four key domains were analyzed: (1) patient selection, (2) index test, (3) reference standard, and (4) flow and timing. Each domain was assessed in terms of its risk of bias, and the first three domains were also assessed in terms of concerns regarding applicability. The risk of bias and concern about applicability for each domain was considered "low," "high," or "unclear." When the answer to questions about risk of bias and applicability was "low" risk or "low" concern, one point was awarded for each item. Articles were grouped into categories of high quality (6 and 7 points), moderate quality (4 and 5 points), and low quality (0-3 points). Additionally, we performed a critical appraisal of the articles guided by the Critical Appraisal Skills Program (CASP) tools (https://casp-uk.net/casp-tools-checklists/). We assessed the study's validity, its results, and their applicability.

## RESULTS

**Figure 1:**
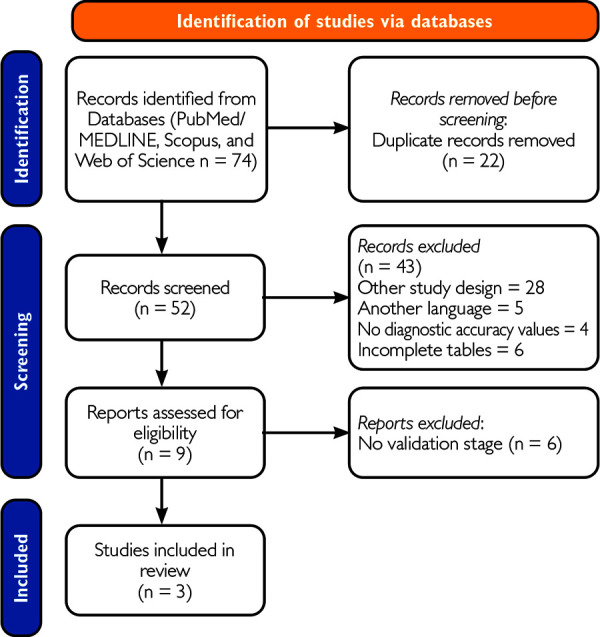
PRISMA Flowchart. A diagram representing the systematic literature search for salivary biomarkers for the diagnosis of gastric cancer patients.

Article selection. The systematic search provided 74 articles, of which we excluded 22 duplicates and 42 for not meeting the eligibility criteria (Figure 1). We obtained 9 articles for full-text review. Among these, 6 articles were excluded as they lacked validation. Finally, we selected 3 articles that met the inclusion criteria.

Study characteristics. The characteristics of the studies are presented in Table 2. These articles, conducted in Korea and China, were published between 2016 and 2018. They used unstimulated whole saliva as the data source. We identified three different diagnostic methods: enzyme-linked immunosorbent assay (ELISA), saliva microarrays, lectin transfer analysis, and quantitative reverse transcription polymerase chain reaction (RT-qPCR). Sample sizes for gastric cancer and non-cancer subjects ranged from 20 to 100 participants. One of the studies also reported a sample of patients with atrophic gastritis (a potentially malignant lesion).

**Table 2:** Key characteristics of included studies.

Study	Country	Ethnicity	Saliva specimen	Method	Sample size		Atrophic gastritis (AG)
					Gastric cancer (GC)	No-cancer (controls)	
Xiao et al., 2016 ^15^	Korea	Asian	Unstimulated whole saliva	ELISA	20	20	No reported
Shu et al., 2017 ^16^	China	No reported	Unstimulated whole saliva	Saliva microarrays and lectin blotting analysis	23	30	24
Li et al., 2018 ^17^	Korea	Asian	Unstimulated whole saliva	RT-qPCR	100	100	No reported

Diagnostic capacity measures in the validation phase. We found salivary biomarkers of extracellular RNA, glycoproteins, and proteins. In the case of glycoproteins, they were used both individually and in combination. Overall, all biomarkers exhibited high diagnostic capacity measures. However, the combination of CSTB, TPI1, and DMBT1 proteins demonstrated the highest AUC value, indicating superior discriminative capacity between gastric cancer patients and healthy individuals. Regarding sensitivity and specificity, values ranged from 70 to 92 and 72 to 91, respectively. Diagnostic capacity measures are detailed in Table 3.

**Table 3:** Diagnostic capacity measures in the validation phase.

Study	Type of biomarker	Biomarker name	Unique combined	or	Accuracy (ROC curve)	Sensitivity	Specificity	P value
Xiao et al., 2016 ^15^	Protein	CSTB, TPI1, DMBT1	Combined		93	85	80	<0,05
Shu et al., 2017 ^16^	Lectin	VVA and SBA	Combined		89 (80-99)	96	80	<0,001
Shu et al., 2017 ^16^	Lectin	VVA	Unique		81 (71-91)	70	91	<0,001
Shu et al., 2017 ^16^	Lectin	LEL and DSA*	Combined		83 (71-94)	92	72	<0,001
Li et al., 2018 ^17^	Extracellular RNA	3 mRNAs (SPINK7, PPL, and SEMA4B) and 2 miRNAs (miR140-5p and miR301a)	Combined		81 (72-89)	75	83	0,093

Lectins are carbohydrate-binding proteins that discriminate between glycopatterns of glycans based on subtle differences in structure. Several lectins, including VVA, LEL and SBA, are generally used to study altered glycans structures in gastric cancer. *Combined biomarkers for an atrophic gastritis diagnostic model. Serine peptidase inhibitor Kazal type 7 (SPINK7), periplakin (PPL), and semaphorin 4B (SEMA4B).

**Figure 2:**
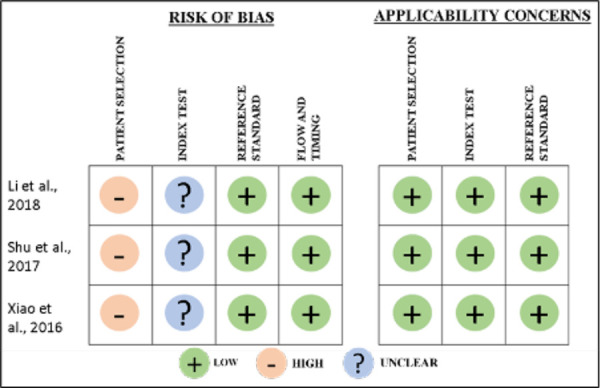
Quality assessment summary (QUADAS-2) of all articles. Quality assessment of comparative diagnostic accuracy studies was used for Xiao et al., 2016 ^15^, Shu et al., 2017 ^16^ and Li et al., 2018 ^17^.

Quality assessment. The QUADAS-2 tool was employed to assess the risk of bias in the studies, covering four key domains: patient selection, index test, reference standard, and flow and timing. The symbol (-) indicates a high risk of bias, the symbol (+) a low risk of bias, and the symbol (?) suggests an unclear risk of bias. In the risk of bias assessment, the most concerning aspect was the lack of information concerning patient selection and unclear details regarding the index test. Consequently, the assessed articles presented moderate quality (Figure 2).

## DISCUSSION

In recent years, various biomarkers in fluids like human saliva have been investigated. This is of significant interest because these biomolecules could contribute to the early diagnosis of diseases such as gastric cancer, which has an unfavorable prognosis due to late diagnosis.

In this study, we identified three types of salivary biomarkers: extracellular RNA (SPINK7, PPL, SEMA4B, miR140-5p, miR301a), carbohydrate-binding proteins (VVA, SBA, LEL, and DSA), and proteins (CSTB, TPI1, and DMBT1). These salivary biomarkers, whether used individually or in combination, exhibited high diagnostic accuracy, represented by their AUC values, making them potentially useful for the detection of gastric cancer and atrophic gastritis.

All the biomarkers were validated in independent cohorts. Initially, they were identified in a discovery phase. Subsequently, the candidate markers advanced to a validation phase, where external samples were used to evaluate diagnostic accuracy. It is noteworthy that during the validation stage, the biomarker's measurement performance characteristics are assessed, determining whether it provides reproducible and accurate data ^18^. According to Ziegler et al., the development of a biomarker involves several phases before implementation in clinical practice, including initial discovery, external validation, clinical utility, and quantification of cost-effectiveness ^19^. The aim of exclusively including studies with a validation phase was to focus our review on salivary markers that are nearing clinical use.

The most promising diagnostic biomarkers were three proteins used in combination. CSTB, a cysteine protein whose expression is reduced in gastric cancer cells, may contribute to the development of this condition by promoting cell proliferation, enhancing metastasis, and inhibiting apoptosis ^20^. TPI1, an enzyme involved in the glycolytic pathway, is overexpressed in cancers such as esophageal cancer due to increased glycolysis ^21^, which provides energy for tumor cell growth ^22^. DMBT1, a complex molecule involved in innate immune defense and epithelial cell differentiation, is overexpressed in gastric cancer, as inflammation is a key factor in metaplastic progression in the stomach ^23^. These three biomarkers were validated using ELISA, a widely adopted method known for its ease and speed ^24^, as in the case of the BioMérieux VIDAS® HIV Panel, a kit for rapid and precise HIV detection. One possible reason for identifying these biomarkers is the presence of exosomes, defined as extracellular vesicles that play a significant role in intercellular communication ^25^, originating from various cell types, including tumor cells. These exosomes contain diverse molecular content, such as proteins or RNA ^26^, and are transported over long distances to be eventually found in bodily fluids like saliva ^27^. The potential of these proteins is so significant that they have been included in a panel of biomarkers patented by David T. Wong in 2018 (US20180188256A1). According to the author, his invention can be used for diagnosing, identifying, or screening individuals with, without, or at risk of having the disease, differentially diagnosing disease states, assessing disease severity or changes in severity, among other potential uses.

The evidence from which these results emerge is of moderate quality. The primary issues in the study designs mainly concern patient selection and the index test. In the selected studies, patients were neither consecutively included nor randomly selected. Additionally, these studies did not avoid a case-control design, which, according to historical arguments, can lead to an overestimation of diagnostic accuracy because the test is evaluated in a group of patients already known to have the disease and in a group of healthy patients ^28^. Furthermore, the studies did not make it clear whether blinding was employed when interpreting the results of the index test, meaning the results of the reference test were not known. This can increase agreement between both tests, resulting in an overestimation of diagnostic capacity measures such as sensitivity and specificity ^29^. Future research should address and rectify these identified deficiencies, especially considering that despite the promising findings, saliva is not yet routinely used as a biological fluid for the detection of gastric cancer.

## CONCLUSION

This systematic review underscores the burgeoning potential of salivary biomarkers in the early detection of gastric cancer, a malignancy characterized by its aggressive nature and poor prognosis when diagnosed at advanced stages. The findings of our review, which focused on studies in their validation phase, reveal significant advancements in the identification and validation of salivary biomarkers, particularly the proteins CSTB, TPI1, DMBT1. These biomarkers exhibited remarkable accuracy in distinguishing gastric cancer patients from healthy individuals, as evidenced by high sensitivity and specificity values. 

The application of non-invasive diagnostic methods like saliva testing offers a transformative approach to cancer diagnosis, aligning with the growing demand for patient-friendly, cost-effective, and accessible healthcare solutions. The ability to detect gastric cancer through salivary biomarkers not only paves the way for early intervention but also minimizes the reliance on more invasive, uncomfortable, and resource-intensive diagnostic methods like endoscopy. The progression of these biomarkers from laboratory research to clinical application will require collaborative efforts across disciplines, underscoring the importance of interdisciplinary research in advancing cancer diagnostics.
